# An Investigation into the Prevalence of *Clostridioides difficile* in Irish Pig Abattoirs and Pork Meat Products as a Potential Source of Human Infection

**DOI:** 10.3390/antibiotics14020151

**Published:** 2025-02-04

**Authors:** Aoife Doyle, Thomas R. Rogers, Declan Bolton, Catherine M. Burgess, Paul Whyte, Jesus Frias, Séamus Fanning, Máire C. McElroy

**Affiliations:** 1 Discipline of Clinical Microbiology, Sir Patrick Dun Research Laboratory, Trinity College Dublin, St. James’s Hospital Campus, D08 RX0X Dublin, Ireland; rogerstr@tcd.ie; 2 Department of Agriculture, Food, and the Marine Laboratories, Backweston, Celbridge, W23 X3PH Kildare, Ireland; 3 Teagasc, Food Research Centre, Ashtown, D15 KN3K Dublin, Ireland; declan.bolton@teagasc.ie (D.B.); kaye.burgess@teagasc.ie (C.M.B.); 4 UCD-Centre for Food Safety, School of Public Health, Physiotherapy & Sports Science, University College Dublin, D05 N2E5 Dublin, Ireland; paul.whyte@ucd.ie (P.W.); sfanning@ucd.ie (S.F.); 5 Sustainability and Health Research Hub, School of Food Science and Environmental Health, Technological University Dublin, Cathal Brugha Street, D01 HV58 Dublin, Ireland; jesus.frias@tudublin.ie

**Keywords:** *Clostridioides difficile*, abattoir, food, pigs, one health

## Abstract

*Clostridioides difficile* (*C. difficile*), once considered a predominantly nosocomial pathogen, is increasingly implicated in community-acquired infections (CA-CDIs). This study investigates the prevalence, ribotypes, and antimicrobial susceptibility of *C. difficile* in Irish pork products and abattoirs, with a focus on the potential public health implications. A total of 180 retail pork products and 150 pig carcase swabs from three abattoirs were examined, alongside 30 environmental lairage samples. The *C. difficile* isolates were characterised through ribotyping and tested in terms of antimicrobial susceptibility. No *C. difficile* was isolated from the retail pork, while the carcase swabs yielded a low recovery rate (0.66%). However, the lairage areas were contaminated with *C. difficile* (33%), and six different ribotypes were identified, including the clinically relevant RT078. The ribotypes exhibited susceptibility to the antibiotics used to treat *C. difficile* infection (CDI) (fidaxomicin, vancomycin, and metronidazole) but showed resistance to tetracycline (9%) and ciprofloxacin (100%). These findings align with the international findings on antimicrobial resistance in *C. difficile* and suggest that strict EU food safety standards could mitigate retail pork contamination risks. Nevertheless, the environmental exposure during slaughtering and handling processes presents potential transmission risks for workers.

## 1. Introduction

*Clostridioides difficile* (*C. difficile*) has historically been regarded as a nosocomial infection, primarily associated with hospital environments due to poor infection control practices. However, recent studies have highlighted that *C. difficile* can be detected in diverse sources beyond healthcare settings, which may contribute to the rise in community-acquired *C. difficile* infections (CA-CDIs) globally [1,2]. Unlike hospital-acquired CDI (HA-CDI), CA-CDI cases often occur in patients who lack the traditional risk factors, such as recent hospital stays, prior antibiotic exposure, or advanced age. In Ireland, one-third of all CDI cases are reported to originate in the community [3].

Certain toxigenic strains, such as ribotype 078 (RT078), are more frequently implicated in CA-CDI, particularly in younger patients, who may carry this pathogen asymptomatically [4]. This finding suggests that these strains have unique phenotypes facilitating their adaptation and subsequent spread outside traditional healthcare settings.

*C. difficile* has been widely detected in livestock and companion animals and may be a potential reservoir for *C. difficile* outside its human hosts, which raises concerns for public health [5,6]. These include pigs, cattle, poultry, household pets such as cats and dogs, animals in slaughterhouses, and various meats and fresh produce [7,8,9,10,11,12,13,14]. Moreover, studies have demonstrated that similar PCR ribotypes are found in both human and animal populations [15,16,17]. Notably, RT078 has been shown to have significant reservoirs in Europe and North America and is a dominant strain in food chain animals, including pigs, regardless of factors such as age, diarrhoeal status, and farm-specific conditions [10]. This strain has been associated with both symptomatic and asymptomatic colonisation in piglets, in cases of typhlocolitis as well as in clinically healthy animals [18,19].

Whole genome sequencing (WGS) has provided compelling evidence of the clonality of porcine and human *C. difficile* isolates, even when human cases are geographically separated [20,21]. While epidemiological links have not been established, the food chain and environmental interactions are potential factors facilitating the dissemination of *C. difficile* across large geographical areas.

There has been an increased emergence of the hypervirulent strains in humans that are commonly detected in animals [18,22]. For example, CA-CDI is commonly associated with RT078, which is also the most common ribotype found in animals, including pigs [23,24]. *C. difficile*’s prevalence is generally higher in farmed pigs compared to other animal species, although the underlying reasons for this disparity are not yet fully understood [9,25]. However, the intensive farming practices commonly associated with commercial pig production may increase the opportunities for pathogen transmission [26].

Commercial pig production is also characterised by high antibiotic use, and the pig industry is a major consumer of these antimicrobials both in Ireland and internationally [27,28]. Antimicrobial administration in piglets often occurs during the weaning stage, when the animal transitions from sow’s milk to solid feed [29]. This coincides with a time when the prevalence of *C. difficile* in piglets is known to be high [6,30,31,32,33]. This raises concerns about the potential for the development of antibiotic resistance.

The overuse of antibiotics drives antibiotic resistance, and pig production is the highest consumer of veterinary antimicrobials in Ireland [28,34]. Due to the high risk of antimicrobial resistance, the WHO has defined macrolides, fluoroquinolones, and cephalosporins as the highest priority, as critically important antimicrobials for human medicine, which should be restricted as part of antimicrobial stewardship programmes [35]. However, the pig industry is a major consumer of these antimicrobials both in Ireland and internationally [27,28].

The primary objective of this study was to perform an initial investigation into the prevalence of *C. difficile* in pig abattoirs and pork meat products within Ireland with a view to gaining insights into the potential of pork meat as a source of human CDIs. The isolates were subsequently characterised by ribotype, and antimicrobial susceptibility.

## 2. Results

### 2.1. Detection of C. difficile in Pig Abattoirs and Meat Products

#### 2.1.1. Detection of *C. difficile* in Meat Product Samples

*C. difficile* was not isolated from any of the 180 retail pork products.

#### 2.1.2. *C. difficile* Recovery from Abattoir Samples

*C. difficile* was detected in environmental swabs taken from the lairage areas of each of the abattoirs, in 10 out of 30 swabs (33%) (Table 1). In contrast, *C. difficile* was identified in only 1 out of 150 (0.66%) swabs taken from pig carcases (Table 1). Six ribotypes were detected across the samples: RT078, RT413, RT066, RT015, RT002, and RT003. Among these, RT078 and RT015 were found to be more prevalent, with each appearing in two of the three surveyed abattoirs.

### 2.2. Antimicrobial Susceptibility of the C. difficile Isolates

The characterisation of the isolates in terms of PCR ribotyping and antimicrobial resistance is presented in Table 2.

## 3. Discussion

There was no detection of *C. difficile* in the Irish retail pork and pork products tested in this study. These findings align with previous European research, which similarly did not detect *C. difficile* in pork products [39,40,41]. However, the results on the prevalence of *C. difficile* contamination in retail meats have been variable. Reports from Europe indicate contamination rates ranging from 0.7% in Italy [42] to 4.7% in Belgium [43]. Outside of Europe, studies also report varied isolation rates, with Canadian data showing contamination from 1.2% [44] to 12% [45], while U.S. reports range from 11.5% up to 42% [46]. These differences may be attributed to variations in the sampling methodologies and isolation techniques and differences in farm contamination levels and slaughtering and handling practices across regions [47,48].

The low isolation rates observed in EU pork products may reflect the impact of strict EU food safety standards, including hazard analysis critical control point (HACCP) protocols and rigorous microbiological quality control measures [49]. The current study, consistent with others, suggests that Irish pork meat is not a major source of *C. difficile* transmission to humans, indicating that other sources likely contribute to community-acquired CDIs (CA-CDIs).

Although higher *C. difficile* isolation rates have been reported in North America, quantitative studies by Weese et. al. have reported contamination levels of 20–60 spores/g in ground pork [45]. The resilience of spores and their heat tolerance could facilitate foodborne transmission, particularly as the infectious dose remains uncertain and may vary based on individual health status or underlying risk factors [15,50].

Environmental exposure may also play a role in CA-CDI. Factory workers and farmers exposed to *C. difficile* could contribute to community spread. A study by Keessen et. al. found that individuals in direct contact with pigs had high carriage rates (21%) of *C. difficile*, particularly RT078 strains, which were genotypically and phenotypically similar to isolates from farm pigs [19]. This is higher than the 11% carriage rate observed in asymptomatic adults, where about half of the strains are toxigenic [51,52]. Contamination of farm floors, air, and workers’ footwear further supports the role of environmental vectors in *C. difficile* spread, paralleling nosocomial contamination patterns [53].

This study found that *C. difficile* was prevalent in the lairage areas of the slaughter plants, with detection rates between 30 and 40% in the pens, which are cleaned and disinfected several times a day using Virkon™ 1% [*w*/*v*]. Virkon™ is a strong oxidising agent containing the active compound potassium peroxymonosulfate but is not fully sporicidal against *C. difficile* spores on hard, non-porous surfaces [54,55]. Alvarez-Perez et al. also reported the presence of *C. difficile* in disinfected lairage areas (37.5%) and trucks (80%) [24]. These findings underscore the risk of contamination transfer from lairages to the processing environment.

RT078, a notable ribotype, was isolated from the lairages of two abattoirs in this study and one carcase post-processing and has been similarly documented in other research [8,24]. Although the carcase contamination is low in this and other studies, it raises concerns about the potential exposure to workers during handling, posing a risk of asymptomatic carriage and subsequent community transmission. However, it remains unclear whether carcase contamination originates from the lairages or whether it is farm-sourced [8].

The most common ribotypes that were found in this study were RT078 and RT015, which have been identified across Europe [13,17,43,56,57,58]. These ribotypes are also commonly isolated from animals and humans, whether asymptomatic carriers of *C. difficile* or those with symptoms of a CDI [16,25].

The main concern, from the viewpoint of the current antibiotic therapy regimens for CDIs in humans, is the emerging resistance to metronidazole and vancomycin [59,60]. Our results provide some reassurance that the isolates detected in this study were susceptible to fidaxomicin, vancomycin, and metronidazole in vitro, which are the treatment choices for a CDI. This is consistent with the current literature, as reports of *C. difficile*’s resistance to these antibiotics are still rare; however, reduced susceptibility of other ribotypes (RT027 and RT001) to vancomycin and metronidazole was reported in a longitudinal surveillance study in Europe [61].

The pig sector uses more antibiotics than other food-producing animal sectors in several countries, and this accounts for approximately 40% of the total veterinary antimicrobial usage in Ireland [28]. In O’Neill et al.’s study, it was found that tetracyclines are the most used antimicrobial agents in Ireland by weight of active ingredient (55.81%), followed by potentiated sulphonamides (25.22%), penicillin (7.84%), and macrolides (7.84%), with 95.6% of all antimicrobial compound usage being classified as prophylactic. As a result, it was not surprising to find some level of tetracycline resistance in the *C. difficile* isolates in this study, as resistance to tetracyclines has also been increasingly noticed internationally in recent years [62].

Potential contamination points in the slaughter process include scalding, evisceration, and the handling of cutting equipment [63,64]. *C. difficile* spores, known for their long-term survivability, may contaminate equipment, as observed in hospital settings, where contaminated tools have been implicated in outbreaks [65]. While scalding at 60 °C reduces many pathogens, it is ineffective against *C. difficile* spores, which can survive harsh conditions [66]. Therefore, infrequent replacement of scalding tank water may be a contributing factor to carcase contamination. Although contamination points beyond the lairages were not examined in this study, future investigations could shed light on the critical contamination points throughout the processing line. However, despite these concerns, the results of this study did not find evidence to suggest that Irish pork products are a significant source of CA-CDIs.

It is important to acknowledge the limitations of this study. The abattoir survey was based on convenience sampling at a single timepoint in each of the three abattoirs. However, these were three high-throughput abattoirs, and carcases from approximately 9.5% of commercial pig herds over a wide geographic area in Ireland were sampled.

In conclusion, this study did not detect *C. difficile* in Irish retail pork and pork products, which aligns with prior European research. These findings suggest that contaminated pork meat is unlikely to be a major source of community-acquired *C. difficile* infection (CA-CDI) in Ireland. The variability in the contamination rates reported globally likely reflects regional differences in farming practices, slaughter processes, cleaning practices, and food safety standards.

This study also underscores the role of environmental exposure, particularly in lairage areas, as a potential vector of *C. difficile* transmission. However, this study did not extensively explore other environmental vectors, such as vermin, water, or airborne transmission in slaughterhouse facilities, which might contribute to *C. difficile* spread [67].

The *C. difficile* isolates detected in this study demonstrated susceptibility to key antibiotics used to treat CDI. Emerging resistance to commonly used antibiotic treatments is concerning, particularly given the widespread use of antimicrobials in pig farming. Future investigations could prioritise exploring contamination points along the processing line and assessing the broader environmental and occupational exposure risks. Collectively, these efforts will enhance the understanding of the pathways through which *C. difficile* may be transmitted between pigs and humans and inform strategies to mitigate its spread, ensuring the continued safety of pork products and public health.

## 4. Materials and Methods

### 4.1. Collection of the Meat Products for C. difficile Surveillance

A total of 180 meat products were tested for the presence of *C. difficile* (Table 3). Between September and December 2021, 100 Irish pork meat products obtained from retail outlets in high-population areas around Ireland as part of an EU antimicrobial resistance monitoring programme were tested. These included pork belly, pork fillet, back bacon joint, and pork chops. An additional 80 Irish pork meat products were sourced from five major supermarkets in Dublin, which included 50 raw pork meat products (sausages, bacon, medallions, and pork mince) and 30 cooked products (cooked ham and pulled pork). All of these products were of Irish origin. Due to their limited availability, no free-range pork products were included.

### 4.2. Collection of the Abattoir Samples

Pork carcase samples were collected from the chill storage rooms in three major abattoirs in the Republic of Ireland. The selected abattoirs were each visited once in 2023 and had slaughter capacities ranging from 10,000 to 25,000 pigs per week. A total of 30 pig herds spanning 15 counties and representing approximately 9.5% of Ireland’s 316 commercial pig herds with over 500 pigs [68] were included in this study. Swabs were taken from five pigs per herd and from four specific anatomical sites on each carcase (the medial hind limb, lateral abdomen, mid-dorsal region, and jowl) using the sampling protocol for *Salmonella* analyses as per Commission Regulation (EU) 2073/2005 [69]. Gloves were changed between samples, and a single, sterile, pre-hydrated Whirl-Pak^®^ Speci-Sponge^®^ (Nasco, Fort Atkinson, WI, USA) was used per carcase, swabbing 100 cm^2^ of each site using vertical and horizontal scrubbing motions. Two sites were swabbed with one side of the sponge, and the other two sites were swabbed with the other. A total of 150 carcase swabs were collected across the abattoirs.

In addition to the carcase samples, environmental samples were taken at four different points on the floor in each of the 10 lairage pens in the three abattoir facilities immediately after they were vacated by a batch of pigs. A total of 30 environmental samples were collected. Gloves were changed in between samples, and a pre-hydrated Whirl-Pak^®^ Speci-Sponge^®^ (Nasco, Fort Atkinson, WI, USA) was used to swab an area of approximately 100 cm^2^. The same swab was used to swab the four different points in each lairage pen, with the swab being turned over after two points had been sampled. The swabs were placed in the original bag they came in, and these bags were labelled with the sample number, date, and pen number. Samples were stored at 4 °C and processed within three hours.

### 4.3. Culture of C. difficile

The carcase and meat product samples were homogenised in brain heart infusion (BHI) broth using a Seward Stomacher^®^ 400 and incubated anaerobically at 37 °C for five days. A volume of 100 µL was plated onto *Clostridium Difficile* Moxalactam Norfloxacin (CDMN) agar and incubated for 48 h under anaerobic conditions. Positive and negative controls were included in each batch. Presumptive colonies were identified according to the characteristic ground-glass morphology and horse manure odour and then confirmed using MALDI-TOF MS (Bruker Daltonik, Bremen, Germany). Pure cultures were obtained by streaking a single colony from the CDMN plate onto Columbia blood agar (E&O Laboratories, Bonnybridge, UK). Isolates were stored on cryovial beads (Gem Scientific, Batley, UK) at −80 °C for further analysis.

### 4.4. Ribotyping

DNA extraction was performed from the confirmed *C. difficile* isolates using the Instagene™ matrix. The PCR ribotyping followed the protocol from European Centre for Disease Prevention and Control [70]. The primers for the amplification of the *C. difficile* 16S (5′-GTGCGGCTGGATCACCTCCT-3′) and 23S (5′-CCCTGCACCCTT-AATAACTTGACC-3′) rRNA genes were as described by Bidet, and the 16S primer was labelled at the 5′ end using 6-carboxyfluorescein (6-FAM) [71]. The PCR required 12.5 µL of HotStarTaq Mastermix (QIAGEN Ltd., Manchester, UK), 0.25 µL of each primer (10 μM), 10 µL of RNase-free water (QIAGEN Ltd., Manchester, UK), and 2 µL of the template. Negative (water) and positive controls (NCTC 13366 (RT027)) were included. Amplification involved an initial denaturation step (15 min at 95 °C), followed by 24 cycles (1 min at 95 °C, 1 min at 60 °C, 1 min at 72 °C), with a final extension step (30 min at 72 °C).

The amplicon analysis was conducted by adding 2 µL of the PCR product to 9.5 µL of Highly Deionized (Hi-Di) Formamide (Applied Biosystems, Warrington, Cheshire, UK) and 0.5 µL of GeneScan 1200 LIZ Size Standard (Applied Biosystems, Warrington, Cheshire, UK). The samples were analysed on an ABI 3500 genetic analyser using a POP-7™ separation matrix and a 36 cm capillary array. Raw data were obtained (.fsa files) and uploaded to the freely available WEBRIBO database (https://webribo.ages.at/, accessed on 30 January 2024).

### 4.5. Antimicrobial Susceptibility Testing

Susceptibility testing for ciprofloxacin, clindamycin, metronidazole, rifampicin, tetracycline, and vancomycin was performed using Etest^®^ (bioMérieux, Marcy-l’Étoile, France). *C. difficile* was cultured on Columbia blood agar (E&O Laboratories, UK) at 37 °C for 48 h under anaerobic conditions using a Whitley A25 anaerobic workstation. Etest^®^ strips were brought to room temperature at least 30 min prior to their use, and fastidious anaerobic agar with 5% [*v*/*v*] horse blood (FAA-HB) (E&O Laboratories Ltd., UK) plates were dried for 15 min at 15 °C. The isolates were suspended in 0.9% [*v*/*v*] saline to a 1.0 McFarland standard and measured using a DensiCHEK™ Plus Nephelometer (Biomerieux, Chineham, UK). A sterile cotton swab was soaked in the inoculum, and excess fluid was removed by rotating the swab against the tube wall. The swab was spread uniformly over the FAA-HB plate. The plates were dried before applying the Etest^®^ strips. The strips were applied using sterile forceps and incubated at 37 °C for 48 h under anaerobic conditions in stacks of up to five plates. The strains *C. difficile* NCTC 13366 (PCR RT027, fluoroquinolone-resistant strain) and *Bacteroides fragilis* ATCC 25285 (vancomycin-resistant) were included for internal control of the procedure.

Susceptibility to fidaxomicin was ascertained as per Erikstrup et al. [72] and F. Berger’s recommendations (via personal communication). The isolates were suspended in 0.9% [*v*/*v*] saline to a 4.0 McFarland standard and measured using a DensiCHEK™ Plus Nephelometer (Biomerieux, UK). An antimicrobial susceptibility test disc (Oxoid, Hampshire, UK) was placed onto FAA-HB agar. A total of 1 mg of fidaxomicin (Tillotts Pharma, Rheinfelden, Switzerland) was dissolved in 1 mL of Dimethylsulfoxide (DMSO; Merck, Darmstadt, Germany), and 10 µL was pipetted onto an empty disc. The plates were incubated at 37 °C for 48 h under anaerobic conditions.

After 48 h, the MIC values were manually recorded. The MIC was determined as the intersection point where the ellipse of no growth bisected with the Etest^®^ strip or the zone of inhibition for disc diffusion. Accurate readings required an appropriate inoculum density and clear zone edges for semi-confluent growth after 48 h. Breakpoints were interpreted using the EUCAST guidelines or the CLSI criteria when the EUCAST breakpoints were unavailable [36,37,73]. If neither guideline provided breakpoints, the MICs were assessed according to Freeman [38]. The isolates were categorised as susceptible (S), intermediate (I), or resistant (R) following the EUCAST recommendations [36]. Intermediate susceptibility was defined when the MIC matched the clinical breakpoint (mg/L).

## Figures and Tables

**Table 1:** Distribution of *C. difficile* in lairages (holding areas pre-slaughter) and carcases in the chill room of the abattoirs.

Abattoir Code	Positive Swabs (Total Samples)		PCR Ribotypes Obtained (n)
	Lairage	Carcase	
Abattoir 1	3 (10)	0 (50)	078 (2)
			413
Abattoir 2	3 (10)	0 (50)	078
			066
			015
Abattoir 3	4 (10)	1 (50)	002
			003
			015 (2)

**Table 2:** Antibiotic susceptibility phenotype profiles and minimum inhibitory concentration (MIC) values of the isolates tested.

Isolate	RT	Antimicrobial Susceptibility (S), Intermediate Resistance (I) or Resistance (R) in µg/mL						
		VAN ^a^	MRD ^a^	FID ^a^	RI ^c^	TET ^b^	CM ^b^	CI ^b^
Lairage	078	S (0.38)	S (0.064)	S (<1)	S (<0.002)	R (8)	S (1.5)	R (>32)
	078	S (0.38)	S (0.125)	S (<1)	S (<0.002)	I (6)	S (0.25)	R (>32)
	002	S (0.38)	S (0.125)	S (<1)	S (<0.002)	S (0.047)	S (1.0)	R (>32)
	003	S (0.38)	S (0.125)	S (<1)	S (<0.002)	S (0.032)	S (0.38)	R (>32)
	078	S (0.38)	S (0.094)	S (<1)	S (<0.002)	I (4)	S (1.5)	R (>32)
	015	S (0.38)	S (0.125)	S (<1)	S (<0.002)	S (0.023)	S (0.064)	R (>32)
	413	S (0.38)	S (0.19)	S (<1)	S (<0.002)	S (0.047)	S (1.5)	R (>32)
	015	S (0.38)	S (0.19)	S (<1)	S (<0.002)	S (0.047)	S (0.75)	R (>32)
	066	S (0.38)	S (0.125)	S (<1)	S (<0.002)	S (0.023)	S (0.38)	R (>32)
	015	S (0.38)	S (0.19)	S (<1)	S (<0.002)	S (0.032)	S (0.38)	R (>32)
Carcase	078	S (0.38)	S (0.19)	S (<1)	S (<0.002)	I (6)	S (1.5)	R (>32)

Breakpoints were taken from ^a^ EUCAST [36], ^b^ CLSI [37], and ^c^ Freeman [38]. Seven antimicrobial agents were tested: vancomycin (VAN), metronidazole (MRD), fidaxomicin (FID), rifampicin (RI), tetracycline (TET), clindamycin (CM), and ciprofloxacin (CI).

**Table 3:** Meat products that were tested for the presence of *C. difficile*.

Type of Meat	Product	Number
Raw meat	Pork belly	25
	Pork fillet	25
	Back bacon joint	25
	Pork chops	25
	Sausages	15
	Bacon	15
	Medallions	10
	Pork mince	5
Cooked meat	Cooked ham	25
	Pulled pork	5
Total		180

## Data Availability

The raw data supporting the conclusions of this article will be made available by the authors on request.
